# Reduction of Healthcare-Associated Infections by Exceeding High Compliance with Hand Hygiene Practices

**DOI:** 10.3201/eid2209.151440

**Published:** 2016-09

**Authors:** Emily E. Sickbert-Bennett, Lauren M. DiBiase, Tina M. Schade Willis, Eric S. Wolak, David J. Weber, William A. Rutala

**Affiliations:** University of North Carolina, Chapel Hill, North Carolina, USA (E.E. Sickbert-Bennett, L.M. DiBiase, T.M. Schade Willis, D.J. Weber, W.A. Rutala);; UNC Health Care, Chapel Hill (E.E. Sickbert-Bennett, L.M. DiBiase, E.S. Wolak, D.J. Weber, W.A. Rutala)

**Keywords:** hand hygiene, healthcare-associated infections, bacteria, enteric infections, nosocomial infections, compliance, *Clostridium difficile*

## Abstract

Improving hand hygiene from high to very high compliance has not been documented to decrease healthcare-associated infections. We conducted longitudinal analyses during 2013–2015 in an 853-bed hospital and observed a significantly increased hand hygiene compliance rate (p<0.001) and a significantly decreased healthcare-associated infection rate (p = 0.0066).

The association between hand hygiene and infection prevention has long been known (if not always fully accepted) since the time of Semmelweis (*1*). The challenge in healthcare settings is to achieve and sustain high compliance among many disciplines of personnel who interact with patients and their environment. We investigated whether an improvement in hand hygiene compliance from a baseline high level (>80%) to an even higher level (>95%) could lead to decreases in healthcare-associated infections (HAI).

## The Study

In October 2013, University of North Carolina Hospitals, an 853-bed facility, implemented a new hand hygiene program (Clean In, Clean Out; http://news.unchealthcare.org/empnews/handhygiene) in all inpatient areas, after a successful pilot implementation of the program in the pediatric intensive care unit (*2*). Key features were that the focus for observation was simply on cleaning hands upon entering and leaving patient rooms and that all healthcare personnel (including physicians, advanced practice providers, nurses, nursing assistants, hospital unit coordinators, housekeeping, radiology, occupational/physical/recreational therapists, nutrition and food services staff, phlebotomists, and respiratory therapists) were asked to make observations and provide immediate feedback to each other (*3*). Previously, infection preventionists and designated nursing staff on each inpatient unit performed covert observations of hand hygiene compliance according to Centers for Disease Control and Prevention (CDC; Atlanta, GA, USA) indications for hand hygiene (*1*), and compliance reports by location were disseminated quarterly. Comprehensive surveillance for device-associated and non–device-associated HAI was assessed by 4 infection preventionists according to CDC National Healthcare Safety Network case definitions and included all hospital locations and all infections.

We compared hand hygiene compliance data from the last quarter of the covert observations by infection preventionists and designated nursing staff to compliance data from the first month of the new program by using a χ^2^ test. Hand hygiene compliance data were collected at the unit level, and hospital-wide estimates were obtained by averaging all reporting units, weighted by patient-days for each respective unit. We also used a χ^2^ to compare the average historical HAI rate from January 2013 until the implementation of the new program in October 2013 to the average HAI rate during the study period of October 2013–February 2015, after implementation of the new program.

We examined overall longitudinal hand hygiene compliance rates and HAI rates during the new program by using generalized linear models to describe overall trends. To examine the association between HAI and hand hygiene compliance, we used Poisson regression using generalized estimating equations with an exchangeable working correlation matrix using SAS version 9.3 (SAS Institute, Inc., Cary, NC, USA). We used data for hand hygiene compliance and number of overall HAI, HAI with multidrug-resistant organisms (MDRO), and healthcare-associated *Clostridium difficile* infection (HA-CDI) from each nursing unit to estimate the overall association between hand hygiene and HAI rates. An offset of patient-days was used to account for varying levels of time at risk for each unit and month.

During the 17-month study period, >4,000 unique observers made >140,000 observations under the new hand hygiene program. We noted a significant increase in overall hand hygiene compliance rate (p<0.001) and a significantly decreased overall HAI rate (p = 0.0066), supported by 197 fewer infections (Figure) and an estimated 22 fewer deaths (*4*). These reductions resulted in an overall savings of US ≈$5 million (*5*).

**Figure:**
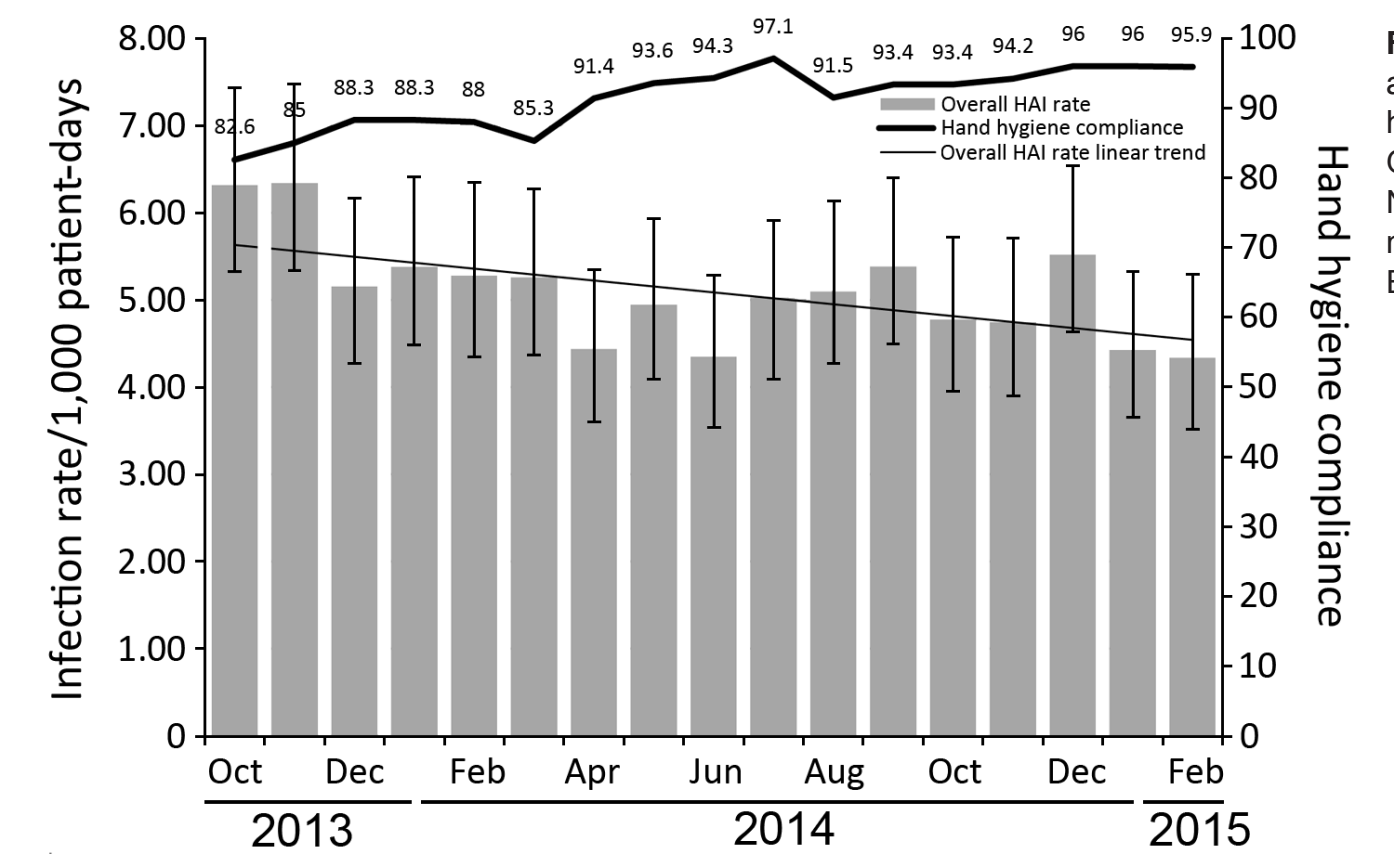
Overall healthcare-associated infection (HAI) rate and hand hygiene compliance by month, October 2013­–February 2015. Numbers above data bar indicate monthly compliance percentages. Error bars indicate 95% CIs.

The association between hand hygiene compliance and HAI, adjusting for unit-level data, showed a 10% improvement in hand hygiene, associated with a 6% reduction in overall HAI (p = 0.086). The association between hand hygiene compliance and HA-CDI, adjusting for unit-level data, showed a 10% improvement in hand hygiene, associated with a 14% reduction in HA-CDI (p = 0.070). No association was noted between hand hygiene compliance and MDRO infections (p = 0.7492).

Hospital-wide hand hygiene compliance measurements by using the previous method (covert observation by designated staff) in the final measurement quarter were not statistically different than in the first month of compliance data measured by all staff in the new program (p = 0.7503). In addition, the average HAI rate in the 9 months before implementation of the new program was not statistically different (p = 0.542) from the average HAI rate during the 17-month study period after implementation.

When the CDC Hand Hygiene Guideline was published in 2002, hand hygiene compliance was summarized on the basis of then-current studies to be very low (average 40%, range 5%–81%) (*1*). Investigators have demonstrated reductions in HAI and MDRO infections when compliance increased from low to medium levels (48% to 66%) (*6*). More recently, hospital epidemiologists and infection preventionists have worked to achieve and sustain higher compliance by using shared accountability, incentives, and feedback strategies (*7*), but until now, no analysis has demonstrated whether an improvement in hand hygiene from a baseline high level (>80%) to an even higher level (>95%) would lead to hospital-wide decreases in HAI (*8*). Demonstrating the importance of continuously improving hand hygiene compliance is critical for staff and hospital leaders who may underestimate the impact on HAI.

Hand hygiene compliance measurements have been studied and methods have been proposed to alleviate concerns associated with interobserver variation, sampling bias, and the Hawthorne effect (*9*). We overcame these concerns by simplifying the compliance measurement to only evaluate the opportunities that cover most (≈87%) of the World Health Organization–defined “Five Moments” on the basis of a 24-hour validation video surveillance of activity in patient rooms; that is, 21% of episodes before patient contact, 22% of episodes after touching a patient, and 44% of episodes after touching patient surroundings (*10*). Furthermore, by engaging all hospital staff in measuring hand hygiene compliance, all opportunities of the hygiene program were eligible opportunities for measurement. In this way, the Hawthorne effect was a consistent presence that became the main intervention for achieving improvement. Finally, the finding that our previous hand hygiene compliance rates measured by trained, designated staff was not statistically different than the compliance rates from the beginning of the new program further supports that the new compliance metric was not affected by any new, unanticipated measurement bias.

Although we cannot eliminate the possibility that other infection prevention factors were also associated with a decreased HAI rate, no other specific hospital-wide infection prevention goals were adopted during the time period of this analysis. The associations (and absence thereof) we found with hand hygiene and specific types of infections are biologically plausible. Absence of association between MDRO HAI and hand hygiene is understandable because many MDRO infections occur in patients who may be colonized before admission, have invasive devices, and are at increased risk for becoming infected with their own flora. However, *C. difficile* infections in healthcare facilities are predominantly spread through contact with infected patients or a contaminated environment, then carried on the hands of healthcare personnel. Therefore, a weak association between HA-CDI reduction and hand hygiene improvement is plausible. Although we adjusted the hand hygiene compliance data by patient-days, some units had patients at much lower risk for infections (e.g., psychiatric units). Despite including units of varying risk for HAI, we demonstrated that increased hand hygiene compliance improvements from already high rates can be an important strategy for achieving infection reductions, particularly for healthcare-associated *C. difficile* infections. 

## Conclusions

A program designed to improve hand hygiene compliance among hospital staff successfully engaged all healthcare personnel in monitoring and improving their own hand hygiene compliance. This pursuit of excellence for hand hygiene compliance led to substantial HAI reductions hospital wide.
